# Supplementary material for: Homo sapiens lithic technology and microlithization in the South Asian rainforest at Kitulgala Beli-lena (c. 45 – 8,000 years ago)
Source: PLoS One. 2022 Oct 13;17(10):e0273450. doi: 10.1371/journal.pone.0273450 (PMC9560501; doi:10.1371/journal.pone.0273450)
Supplement: S2 Fig — (PDF) [file pone.0273450.s002.pdf]

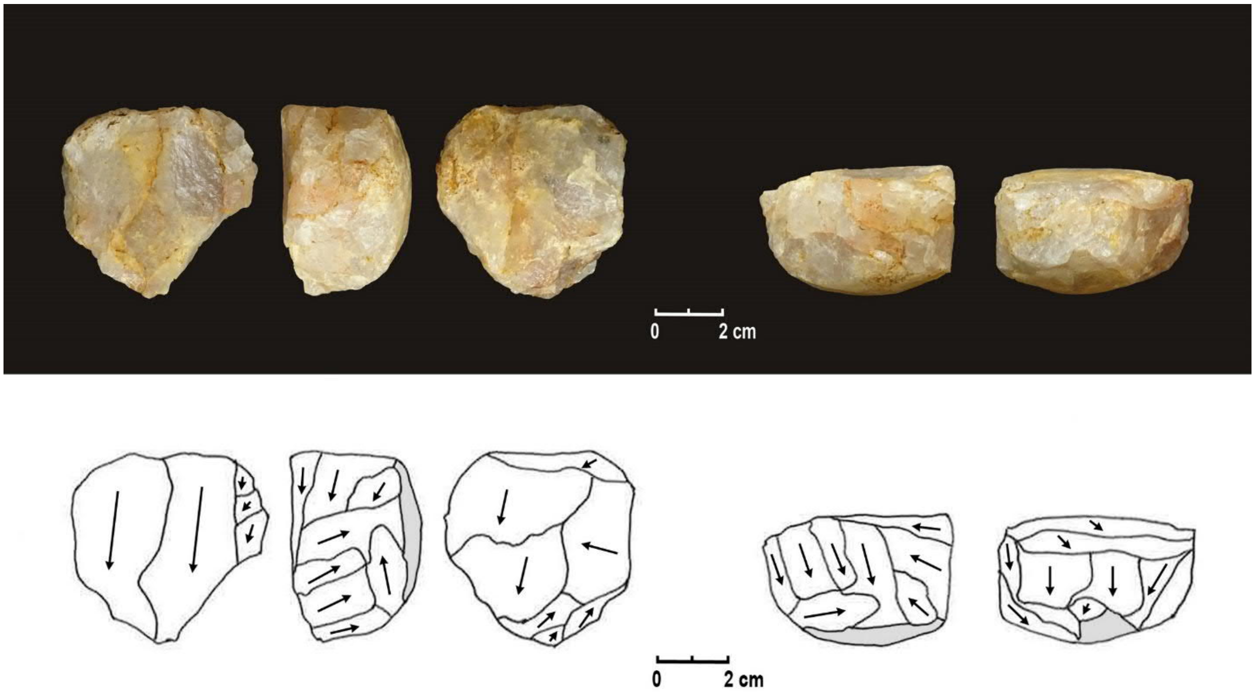


**S2 Fig**: Picture and diacritical scheme of the bipolar anvil-rested from context 19 of Kitulgala Beli-lena.
