# Supplementary material for: Homo sapiens lithic technology and microlithization in the South Asian rainforest at Kitulgala Beli-lena (c. 45 – 8,000 years ago)
Source: PLoS One. 2022 Oct 13;17(10):e0273450. doi: 10.1371/journal.pone.0273450 (PMC9560501; doi:10.1371/journal.pone.0273450)
Supplement: S1 Table — (PDF) [file pone.0273450.s006.pdf]

Phase	Layer	Flake	Bladelet	Fragment	Chips	Core	Core Frag.	Hammer	Total
Late Pleistocene	23	37		62	19	3	2	4	127
	24	7		52	50		1	3	113
	25	14		32	6	2	3	5	62
	26	41		166	158	2	10		377
	22	34		96	51	3	7		191
	21	11		48	41	2	11		113
	39	9		26	22				57
	19	66		426	355	4	5	1	857
	18	5		41	25				71
	17	18		127	205	1		1	352
	16	30		297	220	3	4		554
	13	13		57	40	1	1		112
	15	6		46	28		1		81
	20	11	1	65	90	2			169
	14	1		22	10				33
	12	4		2					6
	38	4		13	24	1	1		43
	35	193	1	533	573	32	19	5	1356
	34	37		214	81	3	2	1	338
Terminal Pleistocene	10	244		1345	1814	48	25	5	3481
	11	7	1	69	56				133
	33	78		484	10	13	12		597
	9	263		737	1350	7	8	1	2366
Holocene	8	206	2	380	313	16	7	1	925
	6	42		338	4	8	6	3	401
	5	23		322	8		1		354
	7	51	1	287	3	2	3	2	349
	27	40		203	94	3	6		346
	4	87	4	274	191	7	6		569
	3	36		317	1	2	2	1	359
	2	37	2	247	6	5	3		300
Total		1655	12	7328	5848	170	146	33	15192

S1 Table: Total number of lithic artefacts by chronological phase at Kitulgala Beli-lena.
