# Supplementary material for: Homo sapiens lithic technology and microlithization in the South Asian rainforest at Kitulgala Beli-lena (c. 45 – 8,000 years ago)
Source: PLoS One. 2022 Oct 13;17(10):e0273450. doi: 10.1371/journal.pone.0273450 (PMC9560501; doi:10.1371/journal.pone.0273450)
Supplement: S2 Table — Categories: Bipolar Vertical; Bipolar Horizontal; Bipolar Horizontal non-axial; Bipolar orthogonal; Bipolar anvil-rested; Bipolar + free hand unidirectional; Free hand unidirectional. (PDF) [file pone.0273450.s007.pdf]

Phase	Layer	Vertical	Horizontal	Horizontal non-axial	Orthogonal	Anvil-rested	Bipolar + uni.	Unidirectional	Total
Late Pleistocene	23	1			2				3
	25	2							2
	26	1			1				2
	22	3							3
	21	2							2
	19	1	1		1	1			4
	17	1							1
	16	2			1				3
	13	1							1
	20	1		1					2
	38				1				1
	35	22	5	2	2			1	32
	34	2	1						3
Terminal Pleistocene	10	35	5	1	5		2	1	49
	33	9		1	2				12
	9	4	1	1				1	7
Holocene	8	8	2	1	4			1	16
	6	6			2				8
	7	2							2
	27	2					1		3
	4	5			2				7
	3	1	1						2
	2	4						1	5
Total		115	16	7	23	1	3	5	170

S2 Table: Total number of cores by chronological phase at Kitulgala Beli-lena. Categories: Bipolar Vertical; Bipolar Horizontal; Bipolar Horizontal non-axial; Bipolar orthogonal; Bipolar anvil-rested; Bipolar + free hand unidirectional; Free hand unidirectional.
