# Supplementary material for: Homo sapiens lithic technology and microlithization in the South Asian rainforest at Kitulgala Beli-lena (c. 45 – 8,000 years ago)
Source: PLoS One. 2022 Oct 13;17(10):e0273450. doi: 10.1371/journal.pone.0273450 (PMC9560501; doi:10.1371/journal.pone.0273450)
Supplement: S3 Table — Values in bold are normally distributed. (PDF) [file pone.0273450.s008.pdf]

	Weight		Length		Width		Thickness	
	Core	Flake	Core	Flake	Core	Flake	Core	Flake
Late Pleistocene	<0.0001	<0.0001	<0.0001	<0.0001	<0.0001	<0.0001	<0.0001	<0.0001
Terminal Pleistocene	<0.0001	<0.0001	**0.02559**	<0.0001	**0.084**	<0.0001	**0.056**	<0.0001
Holocene	0.002	<0.0001	**0.3973**	<0.0001	**0.3702**	<0.0001	**0.5989**	<0.0001

S3 Table: *P* values of Shapiro-Wilk normality test (alpha=0.05) from cores and unbroken flakes by chronological phase at Kitulgala Beli-lena. Values in bold are normally distributed.
